# Rapid increase in atmospheric iodine levels in the North Atlantic since the mid-20th century

**DOI:** 10.1038/s41467-018-03756-1

**Published:** 2018-04-13

**Authors:** Carlos A. Cuevas, Niccolò Maffezzoli, Juan Pablo Corella, Andrea Spolaor, Paul Vallelonga, Helle A. Kjær, Marius Simonsen, Mai Winstrup, Bo Vinther, Christopher Horvat, Rafael P. Fernandez, Douglas Kinnison, Jean-François Lamarque, Carlo Barbante, Alfonso Saiz-Lopez

**Affiliations:** 1 Department of Atmospheric Chemistry and Climate, Institute of Physical Chemistry Rocasolano, CSIC, Serrano 119, 28006 Madrid, Spain; 2 Centre for Ice and Climate, Niels Bohr Institute, University of Copenhagen, Juliane Maries vej 30, Copenhagen Ø, 2100 Denmark; 3 Ca´Foscari University of Venice, Department of Environmental Sciences, Informatics and Statistics, Via Torino 155, 30170 Venice Mestre, Italy; 4 Institute for the Dynamics of Environmental Processes, IDPA-CNR, Via Torino 155, 30170 Mestre, Italy; 5 Department of Applied Mathematics, School of Engineering and Applied Sciences, Harvard University, Cambridge, MA 02138 USA; 6 National Research Council (CONICET), FCEN-UNCuyo, UTN-FRM, Mendoza, 5501 Argentina; 7 Atmospheric Chemistry Observations and Modelling, NCAR, Boulder, CO 80301 USA

## Abstract

Atmospheric iodine causes tropospheric ozone depletion and aerosol formation, both of which have significant climate impacts, and is an essential dietary element for humans. However, the evolution of atmospheric iodine levels at decadal and centennial scales is unknown. Here, we report iodine concentrations in the RECAP ice-core (coastal East Greenland) to investigate how atmospheric iodine levels in the North Atlantic have evolved over the past 260 years (1750–2011), this being the longest record of atmospheric iodine in the Northern Hemisphere. The levels of iodine tripled from 1950 to 2010. Our results suggest that this increase is driven by anthropogenic ozone pollution and enhanced sub-ice phytoplankton production associated with the recent thinning of Arctic sea ice. Increasing atmospheric iodine has accelerated ozone loss and has considerably enhanced iodine transport and deposition to the Northern Hemisphere continents. Future climate and anthropogenic forcing may continue to amplify oceanic iodine emissions with potentially significant health and environmental impacts at global scale.

## Abstract (web summary)

Despite its chemical importance, the evolution of atmospheric iodine concentrations over time is unknown. Here, the authors show that North Atlantic atmospheric iodine levels have tripled since 1950, and propose ozone pollution and enhanced biological production Arctic sea ice thinning as a primary driver.

## Introduction

The biogeochemical cycle of iodine involves ocean emission, atmospheric transformation, new particle formation, uptake on aerosols, heterogeneous recycling, and deposition on land, where iodine enters terrestrial ecosystems^1–3^. Iodine is also a key trace element in the endocrine system of mammals, and is essential for the production of hormones in the thyroid gland. In humans, iodine deficiency causes neurological damage and developmental delays in children, and represents the most common cause of preventable mental retardation^4,5^. Iodine intake by mammals thus represents the last reservoir in the biogeochemical cycle of iodine. Atmospheric iodine is transported from its dominant oceanic source to the continents, where it is adsorbed onto soil and vegetation. Between ocean emission, soil deposition, and ultimate ingestion by animals and humans, iodine participates in a complex variety of physical and chemical atmospheric processes^1^. Laboratory studies have demonstrated the oceanic emission of hypoiodous acid (HOI) and molecular iodine (I_2_) following the deposition of tropospheric ozone (O_3_) to the surface and the subsequent reaction with iodide (I^−^) ions^6,7^. This ocean release of inorganic iodine is estimated to account for 75% of the total source of atmospheric iodine, with the remainder coming from organic iodine (e.g., CH_3_I, CH_2_I_2_, etc.)^8^. Consequently, an increase in global ocean iodine emissions has been hypothesized in response to the human-driven increase in tropospheric ozone levels during the industrial period^9^. The lack of long-term measurements of atmospheric iodine has so far prevented the study of its evolution in the atmosphere.

In this work iodine and sodium were measured in the upper 130 m of the RECAP (REnland ice Cap Project) ice-core spanning the Industrial Period (i.e., 1750–2011), providing an opportunity to understand changes in atmospheric iodine levels in the North Atlantic region and their responses to natural and anthropogenic forcings. We find that although the iodine concentrations do not vary significantly from 1750 to 1950, the abrupt increase observed from 1950 to 2010 is linked to enhanced tropospheric ozone pollution and Arctic sub-ice biological activity.

## Results

### Iodine concentrations in the RECAP ice-core

**Fig. 1:**
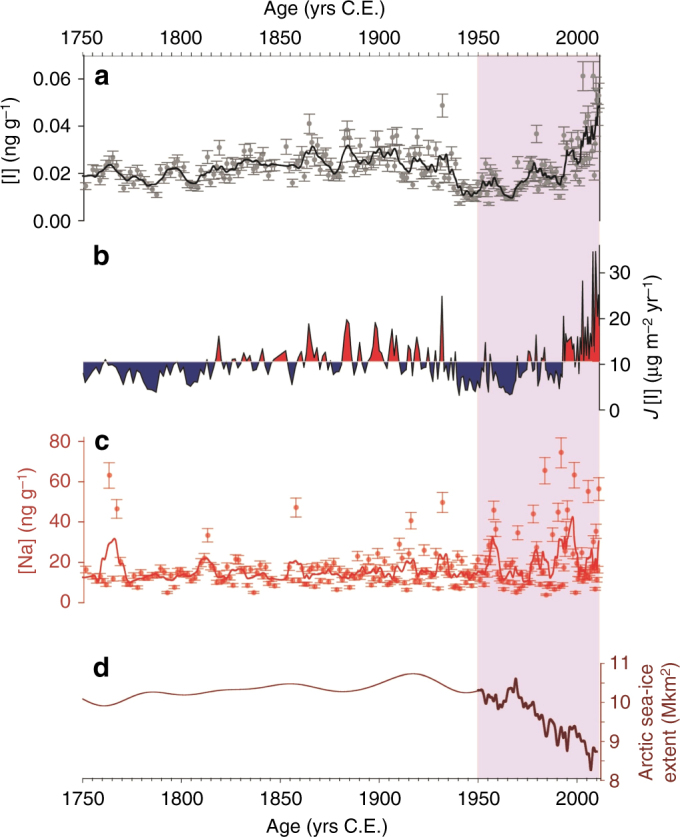
Time series of geochemical elements in the Renland ice-core during the Industrial Period. **a** Iodine [I] concentration and standard deviation, **b** positive (red) and negative (blue) variation of iodine depositional fluxes *J*[I] with respect to the 1750–2010 average and **c** sodium [Na] concentrations and standard deviation from Renland ice core (black and red lines represent the 5-samp. running averages for iodine and sodium, respectively); **d** Arctic sea ice extent reconstruction (thin line from Kinnard et al.^54^ and thick line from Rienecker et al.^55^). The shaded area represents the period 1950–2011 shown in Fig. 2

The Renland ice cap is located on a high elevation plateau on the eastern coast of Greenland (71.30° N, 26.72° W, 2315 m asl) and was the site of a 584-m ice-core drilled to bedrock in 2015. The Renland iodine record displays mean concentrations and deposition fluxes of ~0.023 ng g^−1^ (*σ* = 0.009) and 10 µg m^−^^2^ year^−^^1^ (*σ* = 4), respectively (Fig. 1). Iodine concentrations were stable from the onset of the Industrial Period (1750 Common Era, hereafter C.E.) to 1940 C.E., followed by a drop to values of 0.01 ng g^−^^1^ during the 1940s (Fig. 1). Since the 1950s, iodine concentrations have risen nearly 4-fold, reaching average values of 0.038 ng g^−^^1^ during the past decade (2001–2011). Iodine fluxes have doubled from 1750 to 2010, and tripled from 1950 to 2010.

We now turn to the origin of the observed iodine trend. The Renland ice cap is primarily influenced by the Nordic seas and North Atlantic open waters. There is a weak statistical relationship between iodine and sodium levels, significant at the 90% level, from 1950 to 1989 but not from 1990 to 2011 (Table 1; Supplementary Figure 1). Iodine and sodium are both emitted from the ocean surface, but sodium also has a potential winter emission mechanism via saltation over the sea ice surface^10^. The weak iodine–sodium correlation since 1950 C.E. suggests that the recent iodine variability since 1950 C.E. cannot be attributed to natural processes, such as the atmospheric transport of sea salts. In addition, the iodine–sodium ratio in the Renland ice-core is two orders of magnitude higher than that in seawater^11^ (Supplementary Figure 1).

**Table 1:** Correlation coefficients between iodine concentration and other parameters

	[I]	[Na]	*J*(I)	SST	*E*_sea ice_	O_3_	*B*_prod_ (1979–1989)	Th_sea ice_ (1979–1989)
1950–1989								
[I]								
*ρ*	1	0.296	0.392^*^	−0.35^*^	−0.343^*^	0.362^*^	0.384	−0.278
Sig.		0.075	0.017	0.034	0.038	0.028	0.243	0.408
1990–2011								
[I]								
*ρ*	1	0.207	0.003	0.468^*^	−0.311	0.136	**0.647** ^**^	**−0.677** ^**^
Sig.		0.369	0.991	0.032	0.182	0.567	0.002	0.001

*ρ* Pearson's correlation coefficient, *Sig.* significance (*significance < 0.05, **significance < 0.01 highlighted in bold font)

1950–1989 and 1990–2011 correlation coefficients between iodine concentrations [I] in the Renland ice-core and annually averaged: Renland ice-core sodium concentration [Na], modeled iodine emission fluxes emitted from the North Atlantic Ocean *J*(I), sea surface temperature (SST) in the North Atlantic region^55^, sea ice extent (*E*_sea ice_) in the Arctic region^55^, modeled ozone in the North Atlantic region, biological production^22^ (*B*_prod_), and sea ice thickness (Th_sea ice_) in the Arctic region^22, 23^

**Fig. 2:**
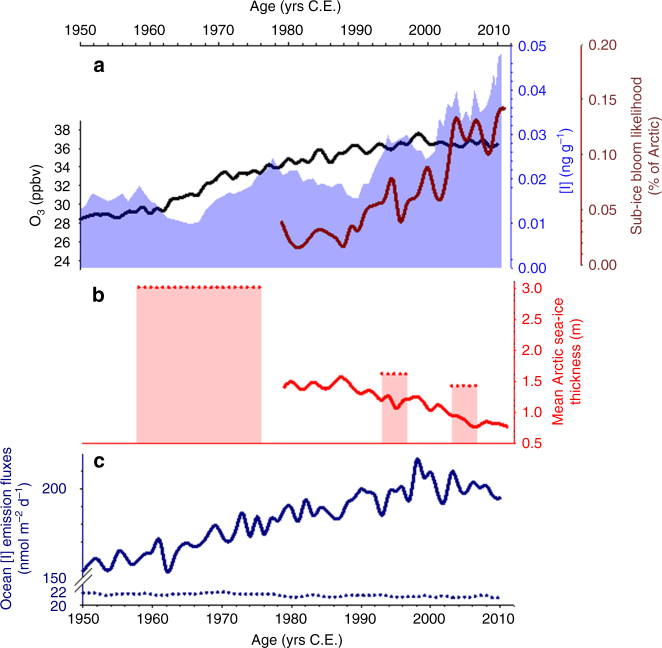
Iodine concentration evolution and forcing mechanisms for the period 1950–2011. **a** Iodine concentration (blue area); ozone annually averaged over the North Atlantic region (latitude: 20° N–70° N, longitude = 75° W–0°) (dark line) and evolution of the pan-Arctic likelihood of sub-ice blooms in late spring and early summer (May–June–July) over time^22^ (red line); **b** mean Arctic sea ice thickness, red line from Horvat et al.^22^ and red dots from Kwok et al.^23^; **c**) modeled ocean emission fluxes of iodine with (solid line) and without (dotted line) the implementation of the ozone-induced iodine emission mechanism

Oceanic emissions of HOI and I_2_, the main source of global atmospheric iodine, depend on surface ozone concentrations^6,7,9^. We note that the sharp increase in iodine concentrations since 1950 has occurred in a period when tropospheric ozone levels have increased globally. The longest quantitative ozone record in Europe indicates that ozone doubled between 1950 and 2000 and has been stable since 2000^12,13^. Results from the chemistry-climate model CAM-Chem (Community Atmospheric Model with chemistry^14,15^; see Methods) show a 33% increase in ocean iodine emissions over the North Atlantic since 1950 in response to a 30% ozone increase during this period (Fig. 2). Over the same period, a baseline simulation without the ozone-induced emission of iodine from the oceans yields a constant iodine emission flux that is 10 times lower than the above results (Fig. 2). During the 1950–1989 period, the iodine concentrations in the ice-core increase significantly with increasing ocean iodine emissions and tropospheric ozone in the North Atlantic (*ρ* = 0.392, *s* = 0.017 and *ρ* = 0.362, *s* = 0.028, respectively (*ρ* = Pearson's correlation coefficient, *s* = significance)). The estimated decreases in ozone precursor emissions over North America and Europe during the 1940s^16^ suggest a similar link during the concurrent observed ice-core iodine decrease. Overall, this suggests that ozone-driven ocean emissions of iodine may have controlled the variability of atmospheric iodine levels during the 1950–1989 period. Note that the increase in atmospheric iodine levels followed by deposition to ice covered areas would in turn make the local heterogeneous processing and recycling of iodine in ice/snow more efficient, as recently measured in the Arctic^17,18^. Interestingly, during the period from 1990 to 2011, iodine concentrations increase despite stable atmospheric ozone concentrations (no significant correlation: *ρ* = 0.136, *s* = 0.567, see Table 1), indicating a different forcing mechanism for iodine emission during the past two decades.

## Discussion

Present-day iodine sources in coastal polar regions are believed to mainly be related with biological production under sea ice^18^ and with abiotic ice surface photochemistry^17,19,20^. The polar amplification of climate change has contributed to a rapid reduction of the Arctic sea ice extent in recent decades (Fig. 1). Arctic sea ice is also increasingly thinning and becoming dominated by thinner and younger sea ice, leading to conditions similar to those found in coastal Antarctica, where the comparatively thinner sea ice allows a more efficient diffusion and release of biologically produced iodine^21^. These recent changes have modified Arctic Ocean ecology by increasing the frequency and extent of sub-ice phytoplankton blooms over the past two decades^22^. We found that iodine concentrations since 1990 are most strongly correlated with the thinning of Arctic sea ice (*ρ* = −0.677; *s*=0.001) and Arctic sub-ice biological productivity (*ρ* = 0.647; *s*=0.002) (Fig. 2; Table 1). Therefore, atmospheric iodine levels in this region may have been significantly influenced by the enhanced sub-ice biological production of iodine since the 1990s. Considering this dynamic, we find no significant correlation between iodine concentrations and sea ice thickness before 1990 (*ρ* = −0.278; *s*=0.408). Thicker Arctic sea ice for the period 1958–1976^23^ (Fig. 2) most likely reduced the ice permeability, hindering the sub-ice biologically related iodine emissions^18,24^. In contrast, since 1990, iodine correlates well with the mean late spring–early summer Arctic sea ice thickness (*ρ* = −0.677; *s*=0.001). The abrupt decrease in the Arctic sea ice thickness below 1.5 m since 1990 (Fig. 2) would ease the propagation of sunlight that ultimately controls the iodine production via algal oxidative stress. This would result in higher iodine excretion rates from Arctic sub-ice algal populations, and a reduced blocking layer due to the more porous fresh ice cap. The high reported values of sea ice thickness before 1960 (Fig. 1) could also account for the decrease in the observed iodine levels during the 1940s and 1950s decades. The attribution to sub-ice Arctic algae blooms is supported by an “off-line” calculation of the resulting iodine flux as a function of seasonal ice fraction, biological activity underneath the sea ice and solar zenith angle. This estimation shows that the contribution of the iodine flux from sub-ice algae to the total iodine emission has increased from 5% in 1960 to 29% in 2010 (Supplementary Note 1). Therefore, the interplay between enhanced biological production and sea ice thinning may explain the higher iodine levels found in the Renland ice-core over the past two decades. Other processes affecting the release and transport of reactive atmospheric iodine in the Arctic, such as the abiotic photochemical recycling occurring on the sea ice surface, are very likely to remain constant in time, or even decrease due to the reduction of sea ice extent since it reduces the active area from which this emission may occur.

A final point to consider is the environmental and health implications of the observed rapid rise in iodine since the mid-twentieth century. Higher iodine levels have led to a 25% larger modeled tropospheric ozone destruction rate in the North Atlantic region since 1950 (Supplementary Figure 2). The modeled surface ozone concentration over the North Atlantic is ~10% higher in the 1950–2010 simulation without the natural O_3_-iodine feedback mechanism. Our modeled iodine deposition rates in Renland are in good agreement with the observations in the ice-core (Supplementary Figure 3). Our modeling results show that transport of marine iodine and its deposition to the North American and European continents have increased by 38% and 25% respectively, during the past 50 years. The enhancement of iodine deposition over continents and the subsequent adsorption onto soil and vegetation^25^ is important since it is estimated that 2 billion people worldwide still have insufficient iodine intake^5,26,27^.

The sustained growth in iodine concentrations in Renland is likely due to human influences on tropospheric ozone and recent global warming in the Arctic. This observation points to a significant increase in the entry of iodine into the ecosystems, which carries important environmental consequences. Enhanced atmospheric iodine levels will likely promote the formation of new ultrafine aerosol particles^28–32^. The acceleration of tropospheric ozone loss due to higher iodine levels leads to a reduction in the oxidative capacity of the atmosphere and a reduction in the ozone radiative forcing. Indeed, at present, the halogen-mediated depletion of tropospheric ozone, a potent greenhouse gas, is estimated to account for 30% of ozone radiative forcing^33–35^. The increase in both the formation of ultrafine particles and ozone destruction leads to a cooling effect on the climate. Atmospheric deposition is also a major source of iodine in soils and plants as it enters the food chain through this mechanism^25^. Therefore, an increasing amount of iodine deposited over the continents since the mid-twentieth century may have led to an increase in human iodine uptake in some regions. Finally, we note that if iodine concentrations continue to increase, they could have significant impacts on future tropospheric ozone, aerosol formation and human iodine intake.

## Methods

### The Renland ice-core

The RECAP ice-core was drilled at 71.30° N, 26.72° W (2315 m asl), on the Renland ice cap in Scoresbysund Fjord (Eastern Greenland) in 2015 using the Danish Hans Tausen intermediate drill system. An ice-core 10 cm in diameter was drilled to bedrock, 584 m below the snow surface. The depth range of the Renland ice-core reported here spans the Industrial Period from a 5.5-m ice-core depth (2011 C.E.) to a 130-m ice-core depth (1750 C.E.). The RECAP ice-core chronology is based on annual layer counting using the StratiCounter algorithm^36^, which accounts for the annual signal in a large array of chemical species^37^—including black carbon, calcium, conductivity, ammonium, sodium, dust, water isotope (d^18^O, dD, deuterium excess), as well as electrical conductivity records (DEP, ECM); in total 17 data series. The timescale was constrained by five prominent volcanic eruption markers within the considered time interval (Hekla 1947 C.E. at a depth of 50.5 m, Katmai 1912 C.E. at a depth of 68 m, Tambora 1816 C.E. at a depth of 106 m, and Laki 1783 C.E. at a depth of 117 m). Given the volcanic constraints, the uncertainty in the age scale is estimated to be ±2 years through the period discussed here. The density profile was obtained by fitting a three-stage exponential densification model (*χ*^2^-fit prob = 7.7%) to density measurements (*N* = 180, *σ*_ρ = _15 kg m^−3^) obtained every 55 cm. The annual accumulation rates were calculated from the annual layer thickness, the density profile and a linear thinning function, which was inferred from a Dansgaard and Johnsen^38^-type ice flow model constrained to well-dated horizons. The average accumulation in the period from 1750 C.E.–present is 447 kg m^−2^ year^−^^1^ (1*σ* = 65 kg m^−^^2^ year^−^^1^), which is consistent with the Holocene value of 50 cm ice equivalent (i.e. 459 kg m^−^^2^ year^−^^1^) reported in a previous work^39^. The ice-core samples were collected in October–December 2015 using a continuous flow analysis (CFA) system at the University of Copenhagen^40^. One discrete sample was collected for each 55-cm rod of melted ice. Meltwater was collected in pre-cleaned polyethylene tubes, subsequently refrozen and stored shielded from light until analysis. Samples were sent to the IDPA-CNR, University Ca’ Foscari of Venice for sodium and iodine determination.

### Iodine quantification

Measurements were carried out at the Environmental Analytical Chemistry laboratory of the IDPA-CNR, University Ca’Foscari of Venice. Samples were measured by collision reaction cell-inductively coupled plasma mass spectroscopy (Agilent 7500cx, Agilent, California, USA) using a Scott spray chamber fitted with a Microflow PFA nebulizer (ESI, Omaha, NE, USA). The operational methodology was carried out as described in a previous work^41^. Iodine (^127^I) and sodium (^23^Na) isotopes were determined, respectively, at a low and medium mass resolution with plasma stability evaluated by the continuous monitoring of ^129^Xe. The sample line was thoroughly cleaned using 2% nitric acid and ultrapure water (UPW, 18.2 MΩ•cm) between each analysis. Instrumental blanks (UPW samples and UPW ice samples from the CFA campaign) were measured throughout the analytical phases. Detection limits of sodium and iodine were respectively 0.8  ppb and 5 ppt—calculated as three times the root mean square of the blank samples. Instrumental-associated errors for iodine and sodium concentration measurements are 10%. Air-to-snow iodine annual fluxes (*J*_I_) were calculated by the following: *J*_I_ = *C*_I_*A*, where *C*_I_ is the iodine concentration in the ice and *A* is the annual accumulation rate.

### CAM-Chem model setup and validation

The model employed in this work is the global 3-D chemistry-climate model CAM-Chem (version 4)^42^, which is included in the CESM framework (Community Earth System Model). The model setup is based on the CCMI-REFC1 experiment described in a previous work^43^, but incorporates an updated halogen chemistry scheme for halogens (chlorine, bromine, and iodine)^14,15,33,44,45^. This model configuration includes an explicit state-of-the-art scheme of iodine emissions (both organic and inorganic) and photochemistry (both gas and particle phase), which explicitly account for chemical transformation during transport from the ocean source to deposition in the Renland region. The iodine chemical scheme includes in addition to a complete gas phase chemical mechanism considering independent photolysis rate coefficients, bi-molecular and termolecular reactions^15^, and a parameterized representation of heterogeneous reactions occurring both on sea-salt aerosols and tropospheric ice crystal^14^, as well as independent representation of dry and wet deposition (including below-cloud wash-out and ice-uptake) for each inorganic iodine species^14,15^. These processes change the chemical partitioning of each of the 14 individual inorganic halogen species by calling the chemical solver coupled with the transport module at each time step.

Halogen sources include the photochemical breakdown of five very short-lived bromocarbons (VSL^Br^ = CHBr_3_, CH_2_Br_2_, CH_2_BrCl, CHBrCl_2_, CHBr_2_Cl) and four iodocarbons (VSL^I^ = CH_3_I, CH_2_ICl, CH_2_IBr, CH_2_I_2_), which are naturally emitted from the ocean to the atmosphere following its production by phytoplankton and photochemical processes occurring at ocean surface^46^. Additionally, abiotic oceanic sources of HOI and I_2_ have been included in the lowest layer of the model^9^, based on recent laboratory studies of the oxidation of aqueous iodide by atmospheric ozone deposited on the ocean surface^6,7^. Therefore, the model includes both organic and inorganic global iodine emission sources.

In this work, CAM-Chem was configured with a horizontal resolution of 1.9° latitude by 2.5° longitude and 26 levels, from the surface to ∼40 km (with eight levels above 100 hPa), as in previous studies^15,44,45^. At the model surface boundary, the zonally averaged distributions of long-lived halocarbons (LL^Cl^ = CH_3_Cl, CH_3_CCl_3_, CCl_4_, CFC-11, CFC-12, CFC-113, HCFC-22, CFC-114, CFC-115, HCFC-141b, HCFC-142b and LL^Br^ = CH_3_Br, H-1301, H-1211, H-1202, and H-2402) based on the A1 halogen scenario from WMO^47^ are considered, while the surface concentrations of CO_2_, CH_4_, H_2_, and N_2_O are specified following a previous work^48^. The model was run in free-running mode^42^ considering prescribed sea surface temperatures (Supplementary Figure 4) and sea ice distributions from 1950 to 2010. Therefore, the model dynamics and transport represent the daily synoptic conditions of the observations, and allows the direct online coupling between the ocean, ice, and atmospheric modules during the 60 years of simulation. To have a reasonable representation of the overall stratospheric circulation, the integrated momentum that would have been deposited above the model top is specified by an upper boundary condition. To evaluate the impact of the increased iodine emissions, all data in the area referred to as the North Atlantic have been averaged between 20° N and 70° N latitude and 75° W–0° longitude. Despite the expected spatial heterogeneities in the oceanic iodine flux due to local changes in SST and wind speed, the increasing trend since the mid-twentieth century is a common feature across the entire ocean.

The model has been extensively compared with available measurements, especially long-term trends of ozone at the surface and in the mid-troposphere^43,49^. While the model shows very good skill for the recent past, there is a tendency for the model simulation to overestimate ozone prior to the 1970s. This would, in turn, reduce the rate of ozone increase over that period, and would therefore underestimate the iodine response to an ozone change. The effectiveness and importance of each reaction and process included in the chemical mechanism has been evaluated in the global atmosphere both during the day and night^50,51^, while all the sources and tropospheric mixing ratios of organic and inorganic iodine species have been validated^8,46^.

The modeled iodine values display a more continuous and less increasing trend than the observed values in the ice-core. The same can be said for the modeled ozone, in comparison to observations from previous works^12,13^. The differences are most likely because there is difficulty in accurately modeling ozone emissions in the past, when measurements were scarce. Furthermore, the model does not implement online the biological activity underneath the sea ice in the Arctic region, and the sea surface production of CH_2_I_2_, CHClI_2_, and CHI_3_ from the reaction of HOI and I_2_ with marine dissolved organic matter^52,53^, although their contribution to the global atmospheric iodine budget is likely to be smaller than that from HOI + I_2_. Therefore, the model is only representative of the ozone-induced inorganic iodine emissions (HOI and I_2_) from the North Atlantic Ocean. Hence, these values must be considered as lower limits, because the modeled trend is lower than that registered in the ice-core. This first difference has been already reported in a previous work^13^, suggesting that in order to reproduce the long-term ozone trends, chemistry-climate models should be improved by introducing additional processes that might have climatic impacts.

### Data availability

The ice-core and model data that support the findings of this study are available upon request.

## Electronic supplementary material

Supplementary Information
