# Supplementary material for: Rapid increase in atmospheric iodine levels in the North Atlantic since the mid-20th century
Source: Nat Commun. 2018 Apr 13;9:1452. doi: 10.1038/s41467-018-03756-1 (PMC5899151; doi:10.1038/s41467-018-03756-1)
Supplement: Supplementary file 1 — Supplementary Information [file 41467_2018_3756_MOESM1_ESM.pdf]

Supplementary information

Rapid increase in atmospheric iodine levels in the North Atlantic since the mid-20th century

Cuevas et al.

Supplementary Figures 1-4

Supplementary Note 1: “Off-line” estimation of Arctic iodine fluxes by biological production

References for Supplementary Information

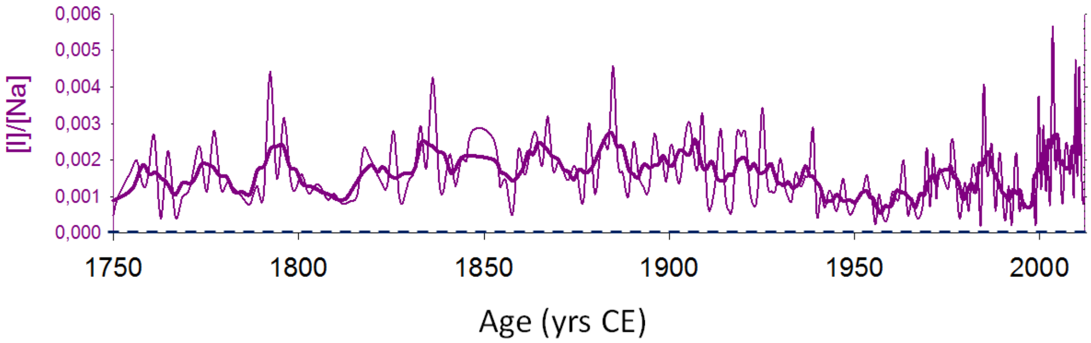

Supplementary Figure 1. **Iodine-sodium ratio in Renland ice core.** Thick line represents the 5 samp. running averages, and dashed blue line represents the average [I]/[Na] seawater ratio[1].

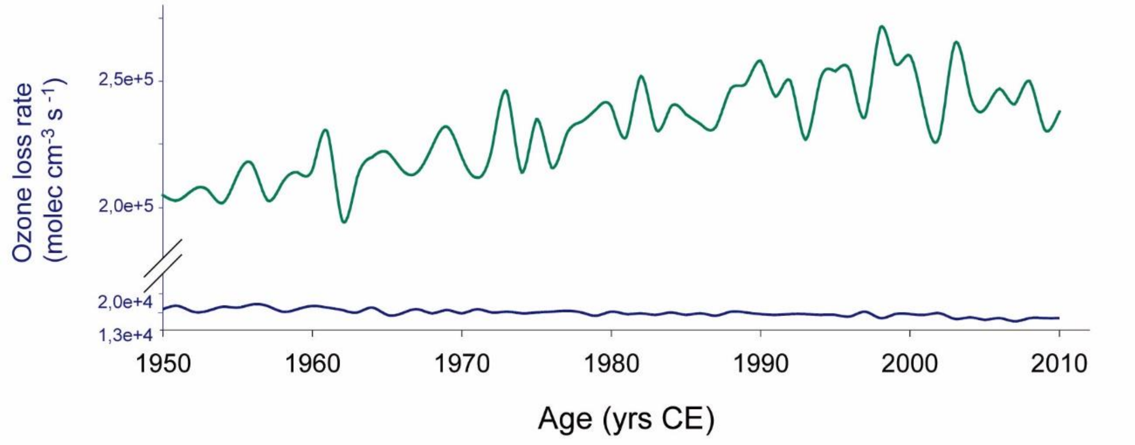

Supplementary Figure 2. **Modelled ozone loss rates due to iodine chemistry**. Loss rates due to active iodine species with (green line) and without (blue line) the implementation of ozone-induced inorganic iodine emissions. Data are averaged over the North Atlantic region (latitude: 20°N-70°N, longitude=75°W-0°).

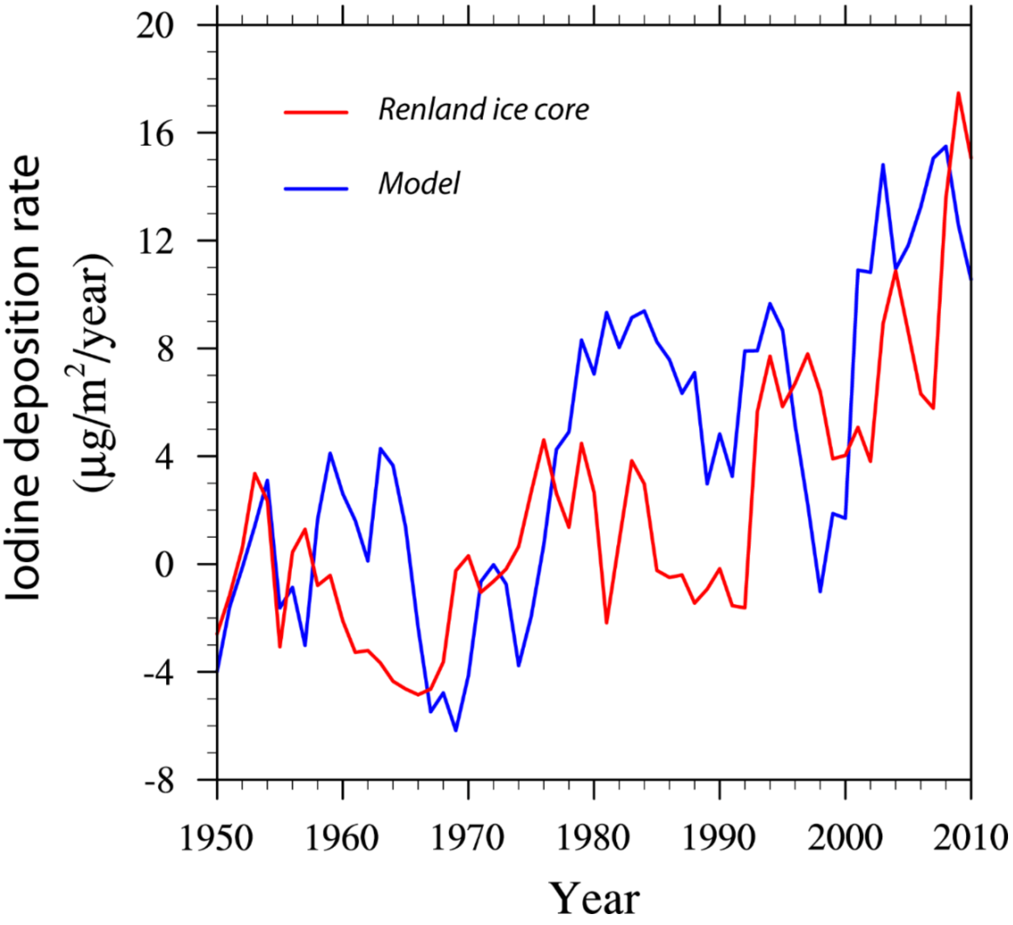

Supplementary Figure 3. Observed (red) and modelled (blue) iodine depositional flux rate at Renland, relative to the 1950-59 average.

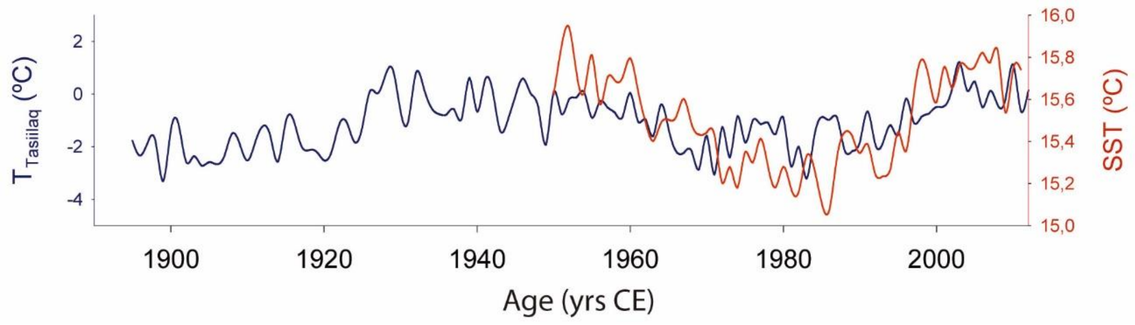

Supplementary Figure 4. **Sea Surface Temperature**. SST obtained from the MERRA retrospective analysis[2] over the North Atlantic region (latitude: 20°N-70°N, longitude=75°W-0°) (red line), and annual temperature record from Tasiilaq meteorological station (65.6°N; 37.61°W, 40 m a.s.l) (blue line).

Supplementary Note 1

"Off-line" estimation of Arctic iodine fluxes by biological production

The estimation of the Arctic iodine fluxes from biological production has been performed by means of an off-line time-slice simulation based on the following equation:

flx_i2_seaice(i) = icefrac(i) * chl_a(i) * cos_SZA(I,t) / max_SZA

where i represents each model gridpoint (lat,lon); icefrac(i) is the modelled sea-ice fraction within each gridbox; chl_a(i) is a monthly dependent chlorophyll-a map used as proxy for the biological activity underneath the sea ice obtained from global satellite observations[https://giovanni.gsfc.nasa.gov/giovanni/#service=TmAvMp&starttime=&endtime=&dataKeyword=NOBM_MON]; cos_SZA(i) is the cosine of Solar Zenith Angle reaching each gridpoint at every timestep (t); and max_SZA is the maximum value for cos_SZA at Renland for each year. The results of two time-slice simulations in the years 1960 and 2010 integrated over the Arctic region (60º-90ºN), considering both the abiotic oceanic source and the sub-sea ice biological source, show that:

1) In 2010 the iodine source from biological blooms underneath the sea-ice accounts for a flux of 57.32 nmol m^{-2} d^{-1}, which represents ~29% of the ozone-induced ocean iodine emissions from the North Atlantic domain (Fig 2c). This would be enough to explain the difference between the 0.3 ppt of IO recently observed in the Arctic [3], and the present-day 0.2 pptv of IO modeled in CAM-Chem at the Renland location.
2) In 1960 de iodine flux from biological blooms is 10.90 nmol $m^{-2}d^{-1}$, which is only ~5% of the ozone-induced ocean iodine emissions from the North Atlantic domain.

Supplementary References

1. Turekian KK. Oceans. *Prentice-Hall, New Jersey*, 120 (1968).

2. Rienecker MM, *et al.* MERRA: NASA's Modern-Era Retrospective Analysis for Research and Applications. *J Clim* **24**, 3624-3648 (2011).

3. Zielcke J. Observations of reactive bromine, iodine and chlorine species in the Arctic and Antarctic with differential optical absorption spectroscopy. PhD dissertation. *Ruperto-Carola University, Heidelberg,* (2015).
